# Supplementary material for: Assessing taxonomic metagenome profilers with OPAL
Source: Genome Biol. 2019 Mar 4;20:51. doi: 10.1186/s13059-019-1646-y (PMC6398228; doi:10.1186/s13059-019-1646-y)
Supplement: Supplementary file 1 — Supplementary tables and figures. (PDF 2601 kb) [file 13059_2019_1646_MOESM1_ESM.pdf]

Additional file 1

Assessing taxonomic metagenome profilers with OPAL

Fernando Meyer, Andreas Bremges, Peter Belmann, Stefan Janssen, Alice C. McHardy, David Koslicki

Table S1. Assessed profiling software, used parameters, and corresponding Bioboxes available as Docker images on Docker Hub.

Software	Parameters	Biobox docker image
CommonKmers	-p <num_cpus> -Q C -k sensitive --normalize	stefanjanssen/docker_profiling_tools:commonkmers
FOCUS CAMI	-m 0.001 -c bd -k 8	stefanjanssen/docker_profiling_tools:focus
Quikr 1.0.0	-n 10 -t SEK	stefanjanssen/docker_profiling_tools:quickr
mOTU 1.1	--processors=<num_cpus> <readfile>	stefanjanssen/docker_profiling_tools:motu
Metaphlan 2.2.0	--mpa_pkl db_v20/mpa_v20_m200.pkl --input_type fastq --nproc <num_cpus> --bowtie2db db_v20/mpa_v20_m200 --bowtie2out /tmp/out.bowtie2 --output_file <output>	stefanjanssen/docker_profiling_tools:metaphlan2
MetaPhyler 1.25	<readfile> blastn <output> <num_cpus>	stefanjanssen/docker_profiling_tools:metaphyler
TIPP 2.0.0	ENV REFERENCE $PREFIX/share/tipp/	stefanjanssen/docker_profiling_tools:tipp

Table S2. Run time in hours and maximum memory usage in gigabytes (in parentheses) required by profilers to process different datasets.

Software	CAMI I high complexity dataset	CAMI II mouse gut dataset	HMP Mock Community dataset
Metaphlan 2.2.0	2.13h (1.44GB)	12.50h (1.66GB)	0.09h (1.41GB)
FOCUS CAMI	2.34h (4.99GB)	13.45h (4.99GB)	0.07h (5.12GB)
Quikr	2.12h (24.12GB)	16.32h (26.12GB)	0.25h (24.62GB)
mOTU 1.1	3.82h (1.19GB)	19.83h (1.19GB)	0.15h (1.01GB)
CommonKmers	11.28h (24.16GB)	76.82h (26.03GB)	0.61h (21.92GB)
MetaPhyler 1.25	31.16h (6.85GB)	119.51h (2.56GB)	0.44h (1.92GB)
TIPP 2.0.0	32.01h (81.53GB)	151.01h (67.51GB)	-

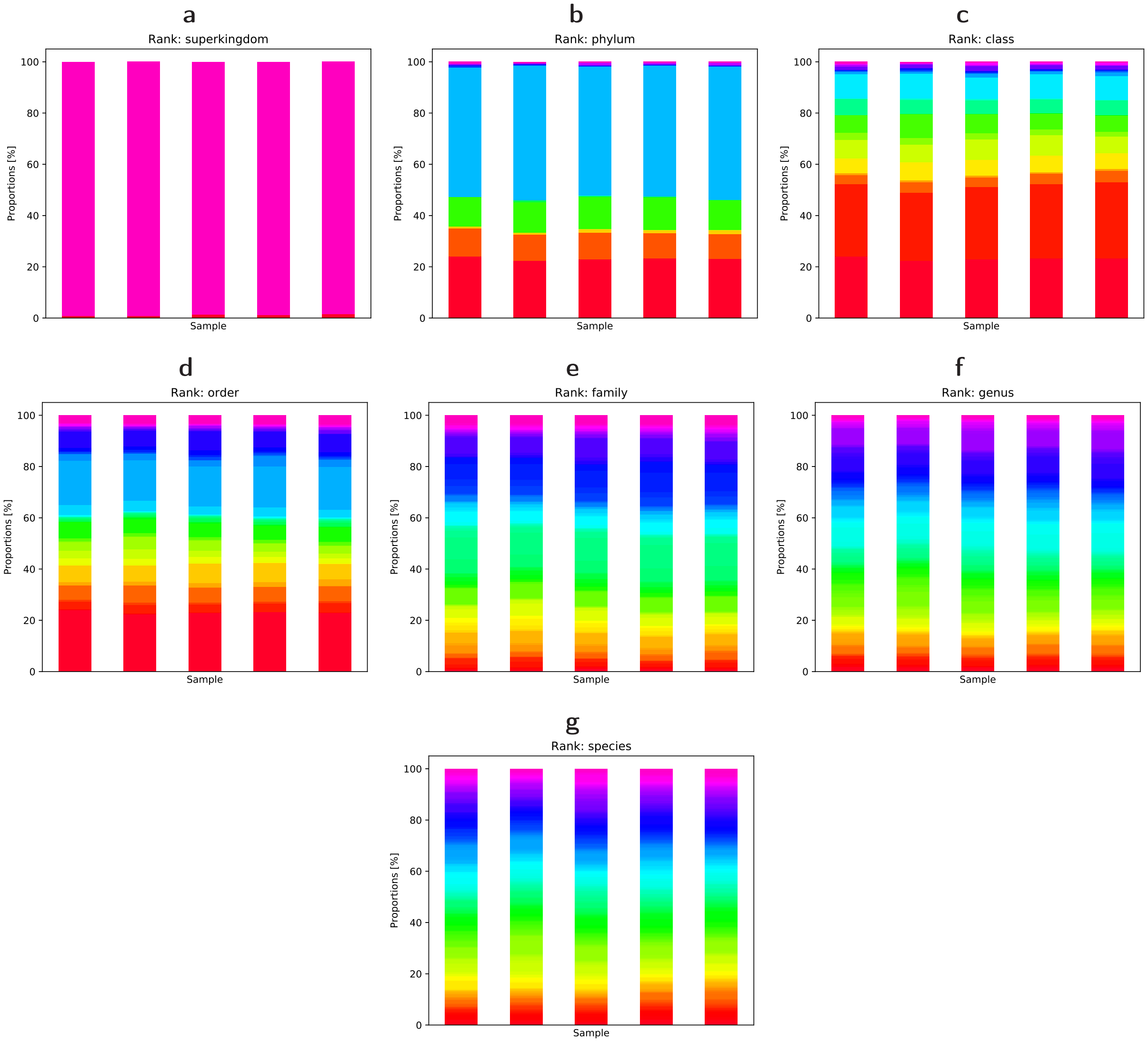

Fig. S1. Taxa proportions per sample for the CAMI I high complexity dataset at different taxonomic ranks. At the superkingdom level, most organisms in all samples are Bacteria, with the proportion of Archea varying between 0.8 and 1.5%.

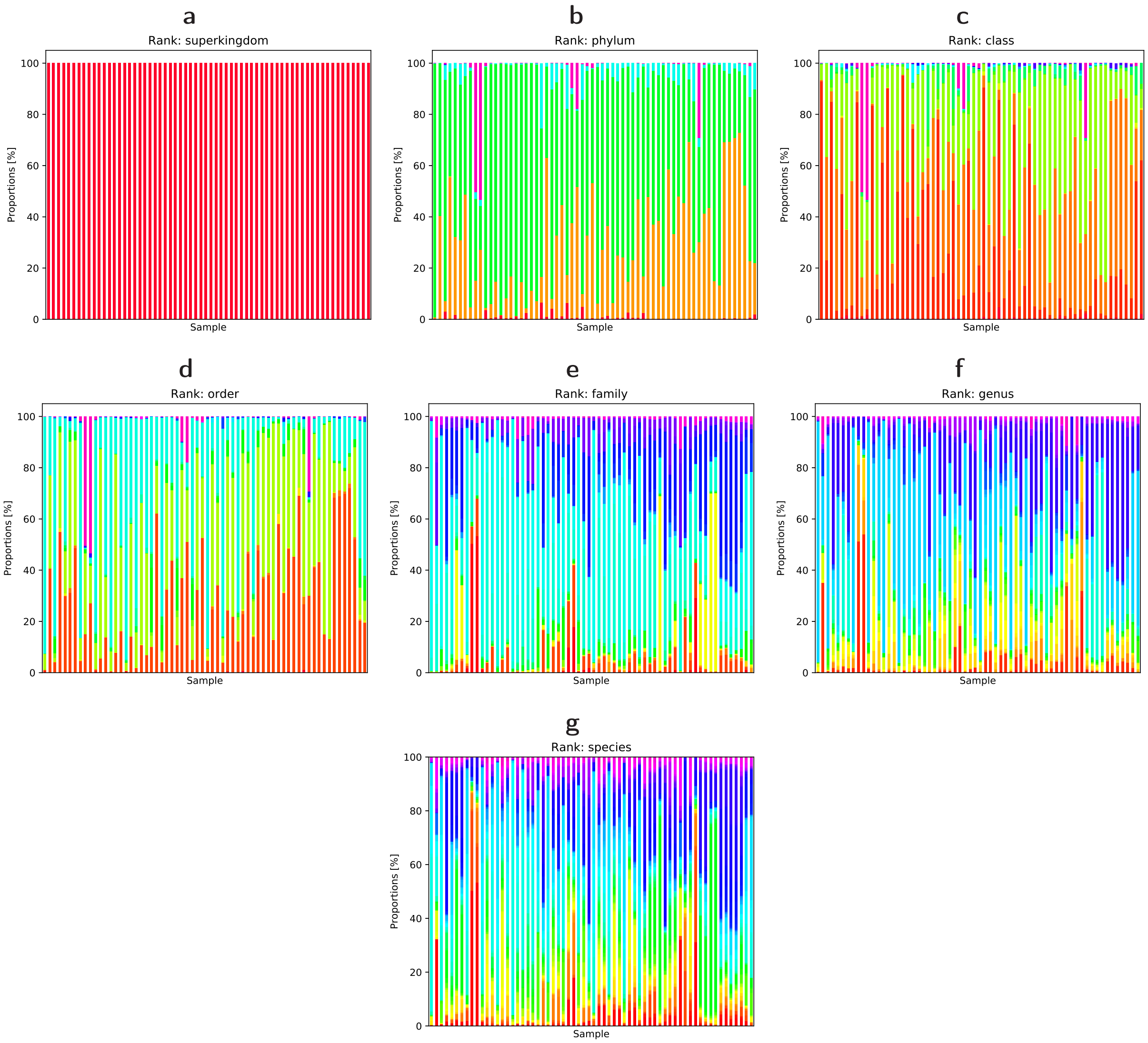

Fig. S2. Taxa proportions per sample for the CAMI II mouse gut dataset at different taxonomic ranks. At the superkingdom level, 100% of the organisms in all samples are Bacteria.

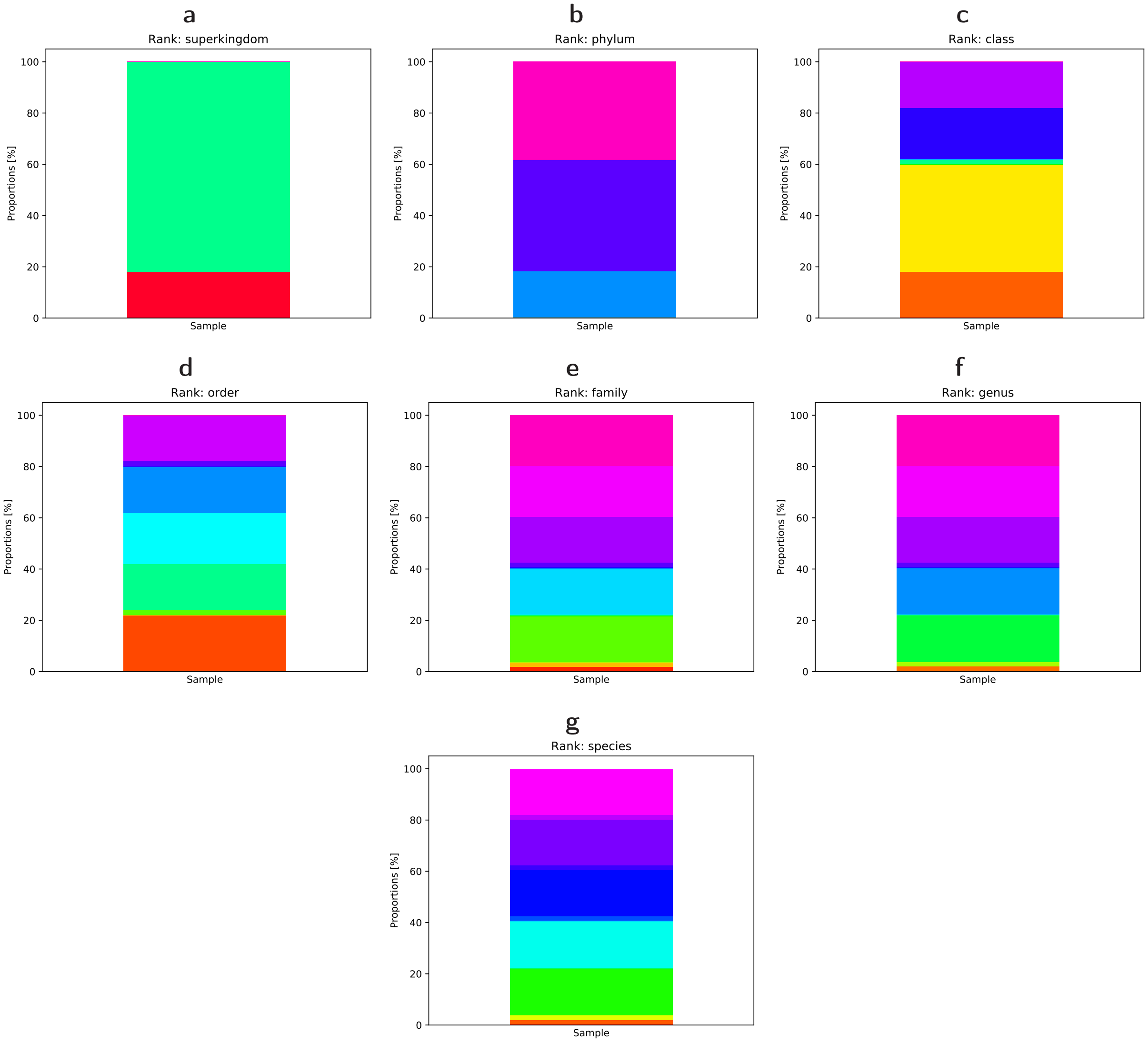

Fig. S3. Taxa proportions for the HMP Mock Community dataset, staggered sample, at different taxonomic ranks. This dataset contains 22 species, with 82.01%, 17.97%, and 0.02% of relative abundance being Bacteria, Archaea, and Eukaryota, respectively.

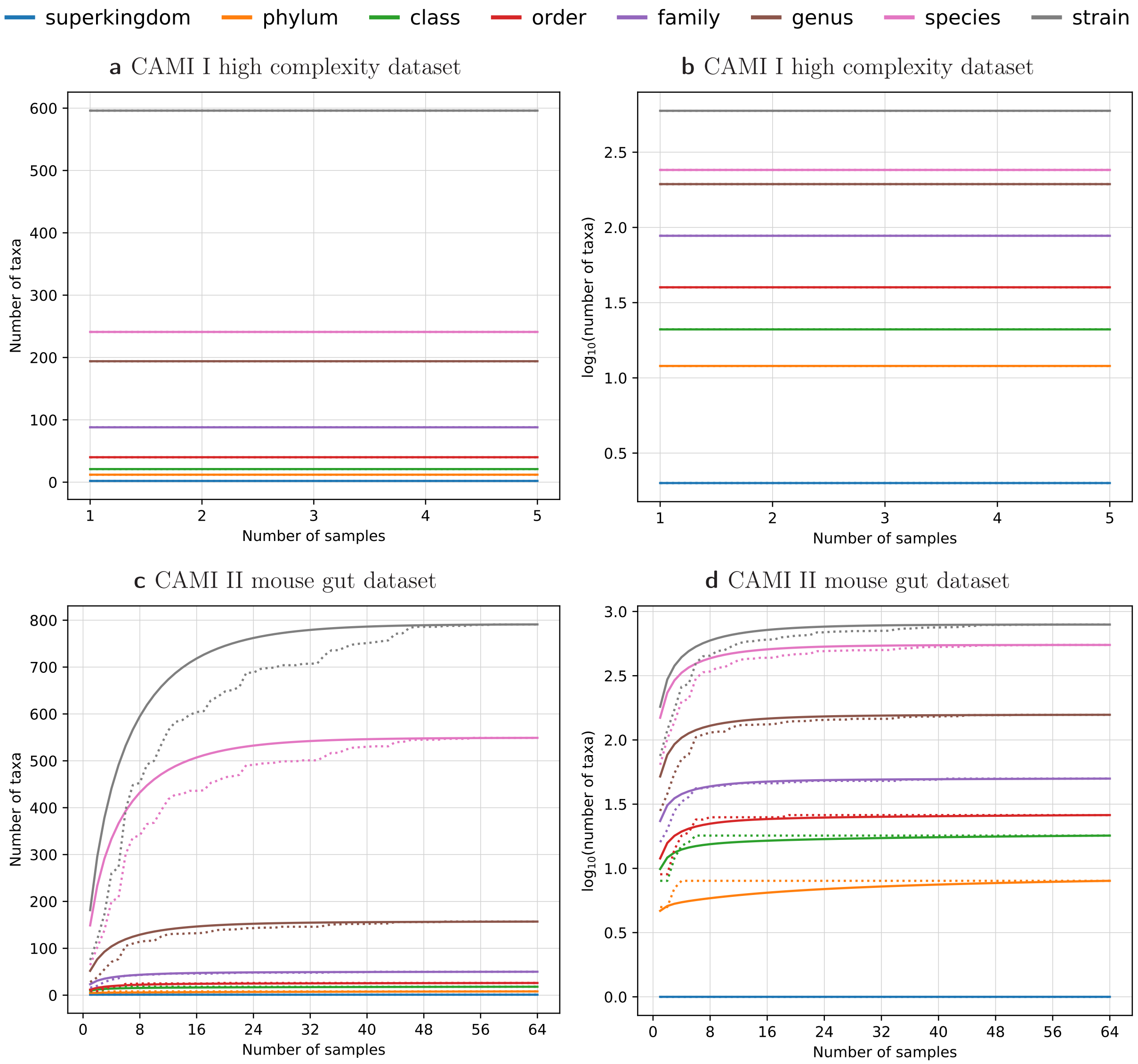

Fig. S4. Rarefaction curves (solid lines) for the CAMI I high complexity dataset (a-b) and the CAMI II mouse gut dataset (c-d) at different taxonomic ranks. Dotted lines are accumulation curves. **b** and **d** are in logarithmic scale with base 10. OPAL always assumes that the samples are from the same environment.

a CAMI I high complexity dataset

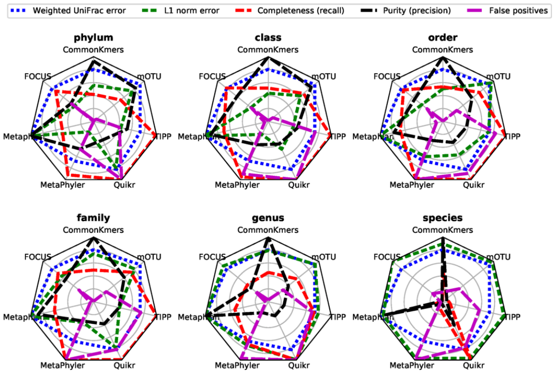

b CAMI I high complexity dataset

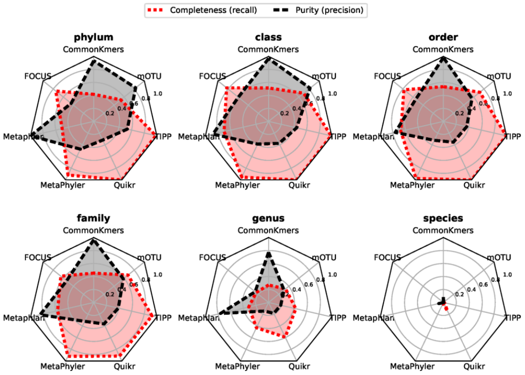

c HMP Mock Community dataset

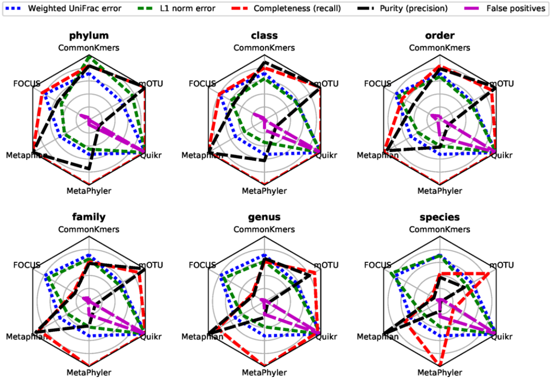

d HMP Mock Community dataset

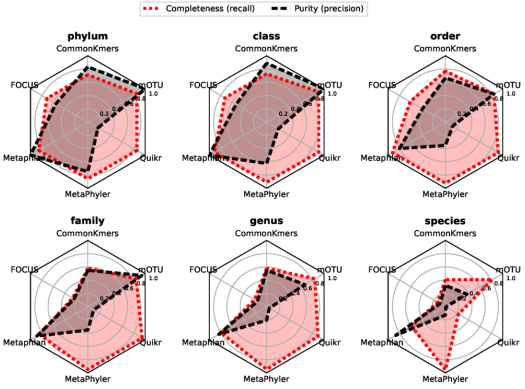

e CAMI I high complexity dataset

	Software (score)				
**Ranking per metric**	**Completeness**	**Purity**	**L1 norm**	**Weighted UniFrac**	**Sum of scores**
1st	Quikr (9)	CommonKmers (4)	MetaPhyler (14)	MetaPhyler (0)	TIPP (184)
2nd	TIPP (31)	Metaphlan (23)	FOCUS (37)	TIPP (5)	MetaPhyler(185)
3rd	MetaPhyler (35)	mOTU (49)	TIPP (52)	CommonKmers (13)	CommonKmers (220)
4th	FOCUS (95)	FOCUS (83)	CommonKmers (59)	Quikr (15)	FOCUS (232)
5th	mOTU (98)	TIPP (96)	Quikr (99)	FOCUS (17)	Quikr (257)
6th	Metaphlan (113)	Quikr (134)	mOTU (116)	mOTU (25)	mOTU (288)
7th	CommonKmers (144)	MetaPhyler (136)	Metaphlan (148)	Metaphlan (30)	Metaphlan (314)

f HMP Mock Community dataset

	Software (score)				
**Ranking per metric**	**Completeness**	**Purity**	**L1 norm**	**Weighted UniFrac**	**Sum of scores**
1st	MetaPhyler (0)	Metaphlan (2)	Metaphlan (2)	Metaphlan (0)	Metaphlan (12)
2nd	Metaphlan (8)	mOTU (3)	MetaPhyler (3)	MetaPhyler (1)	MetaPhyler (20)
3rd	Quikr (8)	CommonKmers (10)	mOTU (11)	mOTU (2)	mOTU (30)
4th	mOTU (14)	MetaPhyler (16)	CommonKmers (17)	CommonKmers (3)	CommonKmers (50)
5th	CommonKmers (20)	FOCUS (19)	FOCUS (17)	FOCUS (4)	Quikr (63)
6th	FOCUS (25)	Quikr (25)	Quikr (25)	Quikr (5)	FOCUS (65)

Fig. S5. Assessment results on the CAMI I high complexity (a, b, e) and the HMP Mock Community (c, d, f) datasets. a, **c** Relative performance plots with results for the metrics weighted UniFrac, L1 norm, completeness, purity, and number of false positives at different taxonomic ranks. The values of the metrics in these plots are normalized by the maximum value attained by any profiler at a certain rank. **b**, **d** Absolute performance plots with results for the metrics completeness and recall, ranging between 0 and 1. **e**, **f** Rankings of the profilers according to their performance and scores for different metrics computed over all samples and taxonomic ranks. For details, see main text.

Worst Median Best

a superkingdom - CAMI I high complexity dataset

Tool	Gold standard	CommonKmers	FOCUS	Metaphlan	MetaPhyler	Quikr	TIPP	mOTU
**Presence/absence of taxa**								
Completeness (recall)	1 (0)	1 (0)	1 (0)	1 (0)	1 (0)	1 (0)	1 (0)	1 (0)
Purity (precision)	1 (0)	0.867 (0.0816)	1 (0)	1 (0)	1 (0)	0.667 (0)	1 (0)	1 (0)
F1 score	1 (0)	0.92 (0.049)	1 (0)	1 (0)	1 (0)	0.8 (0)	1 (0)	1 (0)
True positives	2 (0)	2 (0)	2 (0)	2 (0)	2 (0)	2 (0)	2 (0)	2 (0)
False positives	0 (0)	0.4 (0.245)	0 (0)	0 (0)	0 (0)	1 (0)	0 (0)	0 (0)
False negatives	0 (0)	0 (0)	0 (0)	0 (0)	0 (0)	0 (0)	0 (0)	0 (0)
Jaccard index	1 (0)	0.867 (0.0816)	1 (0)	1 (0)	1 (0)	0.667 (0)	1 (0)	1 (0)
**Abundance estimates**								
Weighted UniFrac error	0 (0)	7.65 (0.0572)	7.79 (0.0963)	9.49 (0.0828)	6.45 (0.0196)	7.81 (0.0582)	7 (0.0352)	8.74 (0.0447)
Unweighted UniFrac error	0 (0)	5.56e+03 (97.8)	5.92e+03 (79.2)	6.27e+03 (39.1)	1.22e+04 (68.3)	1e+04 (148)	7.46e+03 (49.6)	6.48e+03 (68.7)
L1 norm error	0 (0)	0.0241 (0.00431)	0.0388 (0.00654)	0.14 (0.0203)	0.000988 (7.46e-05)	0.0668 (0.00405)	0.0081 (0.000946)	0.258 (0.0373)
Bray-Curtis distance	0 (0)	0.012 (0.00216)	0.0194 (0.00327)	0.0701 (0.0102)	0.000494 (3.73e-05)	0.0334 (0.00203)	0.00405 (0.000473)	0.129 (0.0186)
**Alpha diversity**								
Taxon counts	2 (0)	2.4 (0.245)	2 (0)	2 (0)	2 (0)	3 (0)	2 (0)	2 (0)
Shannon diversity	0.0635 (0.00643)	0.114 (0.0149)	0.138 (0.0086)	0.28 (0.0291)	0.063 (0.00648)	0.214 (0.0032)	0.0807 (0.00758)	0.399 (0.0379)
Shannon equitability	0.0916 (0.00928)	0.134 (0.0128)	0.2 (0.0124)	0.403 (0.0419)	0.0909 (0.00934)	0.195 (0.00291)	0.116 (0.0109)	0.576 (0.0546)

b phylum - CAMI I high complexity dataset

Tool	Gold standard	CommonKmers	FOCUS	Metaphlan	MetaPhyler	Quikr	TIPP	mOTU
**Presence/absence of taxa**								
Completeness (recall)	1 (0)	0.417 (0)	0.75 (0.0456)	0.5 (0)	0.917 (0)	1 (0)	0.983 (0.0167)	0.533 (0.0425)
Purity (precision)	1 (0)	0.933 (0.0408)	0.446 (0.02)	1 (0)	0.474 (0.00399)	0.319 (0.00209)	0.532 (0.00904)	0.828 (0.0605)
F1 score	1 (0)	0.575 (0.008)	0.558 (0.0268)	0.667 (0)	0.625 (0.00349)	0.484 (0.0024)	0.69 (0.00961)	0.645 (0.0428)
True positives	12 (0)	5 (0)	9 (0.548)	6 (0)	11 (0)	12 (0)	11.8 (0.2)	6.4 (0.51)
False positives	0 (0)	0.4 (0.245)	11.2 (0.735)	0 (0)	12.2 (0.2)	25.6 (0.245)	10.4 (0.4)	1.4 (0.51)
False negatives	0 (0)	7 (0)	3 (0.548)	6 (0)	1 (0)	0 (0)	0.2 (0.2)	5.6 (0.51)
Jaccard index	1 (0)	0.404 (0.00785)	0.389 (0.0257)	0.5 (0)	0.455 (0.00367)	0.319 (0.00209)	0.527 (0.0111)	0.482 (0.0454)
**Abundance estimates**								
Weighted UniFrac error	0 (0)	7.65 (0.0572)	7.79 (0.0963)	9.49 (0.0828)	6.45 (0.0196)	7.81 (0.0582)	7 (0.0352)	8.74 (0.0447)
Unweighted UniFrac error	0 (0)	5.56e+03 (97.8)	5.92e+03 (79.2)	6.27e+03 (39.1)	1.22e+04 (68.3)	1e+04 (148)	7.46e+03 (49.6)	6.48e+03 (68.7)
L1 norm error	0 (0)	0.28 (0.018)	0.189 (0.00876)	0.502 (0.0179)	0.0899 (0.00315)	0.398 (0.00892)	0.217 (0.00579)	0.38 (0.0172)
Bray-Curtis distance	0 (0)	0.14 (0.00901)	0.0944 (0.00438)	0.251 (0.00896)	0.045 (0.00158)	0.199 (0.00446)	0.108 (0.0029)	0.19 (0.00859)
**Alpha diversity**								
Taxon counts	12 (0)	5.4 (0.245)	20.2 (0.97)	6 (0)	23.2 (0.2)	37.6 (0.245)	22.2 (0.49)	7.8 (0.583)
Shannon diversity	1.34 (0.0092)	1.03 (0.0248)	1.56 (0.015)	1.02 (0.0308)	1.26 (0.00675)	2.04 (0.0129)	1.18 (0.00576)	1.19 (0.0336)
Shannon equitability	0.541 (0.0037)	0.615 (0.0122)	0.519 (0.00472)	0.571 (0.0172)	0.402 (0.00264)	0.562 (0.00361)	0.358 (0.00175)	0.583 (0.0253)

c class - CAMI I high complexity dataset

Tool	Gold standard	CommonKmers	FOCUS	Metaphlan	MetaPhyler	Quikr	TIPP	mOTU
**Presence/absence of taxa**								
Completeness (recall)	1 (0)	0.514 (0.00952)	0.829 (0.019)	0.705 (0.00952)	0.952 (0)	1 (0)	0.99 (0.00952)	0.695 (0.0323)
Purity (precision)	1 (0)	0.967 (0.0204)	0.597 (0.0159)	0.881 (0.00147)	0.388 (0.00385)	0.375 (0.00474)	0.445 (0.00587)	0.819 (0.0228)
F1 score	1 (0)	0.671 (0.0079)	0.694 (0.0134)	0.783 (0.00654)	0.551 (0.00389)	0.546 (0.00501)	0.614 (0.00655)	0.751 (0.0271)
True positives	21 (0)	10.8 (0.2)	17.4 (0.4)	14.8 (0.2)	20 (0)	21 (0)	20.8 (0.2)	14.6 (0.678)
False positives	0 (0)	0.4 (0.245)	11.8 (0.8)	2 (0)	31.6 (0.51)	35 (0.707)	26 (0.548)	3.2 (0.374)
False negatives	0 (0)	10.2 (0.2)	3.6 (0.4)	6.2 (0.2)	1 (0)	0 (0)	0.2 (0.2)	6.4 (0.678)
Jaccard index	1 (0)	0.505 (0.00891)	0.531 (0.0157)	0.643 (0.0087)	0.38 (0.00371)	0.375 (0.00474)	0.443 (0.00686)	0.605 (0.0332)
**Abundance estimates**								
Weighted UniFrac error	0 (0)	7.65 (0.0572)	7.79 (0.0963)	9.49 (0.0828)	6.45 (0.0196)	7.81 (0.0582)	7 (0.0352)	8.74 (0.0447)
Unweighted UniFrac error	0 (0)	5.56e+03 (97.8)	5.92e+03 (79.2)	6.27e+03 (39.1)	1.22e+04 (68.3)	1e+04 (148)	7.46e+03 (49.6)	6.48e+03 (68.7)
L1 norm error	0 (0)	0.364 (0.00923)	0.285 (0.0206)	0.834 (0.0225)	0.187 (0.00562)	0.398 (0.00738)	0.329 (0.00976)	0.531 (0.0198)
Bray-Curtis distance	0 (0)	0.182 (0.00462)	0.143 (0.0103)	0.417 (0.0112)	0.0935 (0.00281)	0.199 (0.00369)	0.165 (0.00488)	0.265 (0.00989)
**Alpha diversity**								
Taxon counts	21 (0)	11.2 (0.374)	29.2 (0.97)	16.8 (0.2)	51.6 (0.51)	56 (0.707)	46.8 (0.583)	17.8 (0.583)
Shannon diversity	2.16 (0.0136)	1.83 (0.0247)	2.23 (0.0199)	2.01 (0.0353)	2.03 (0.0124)	2.71 (0.00896)	1.91 (0.00945)	1.92 (0.0155)
Shannon equitability	0.709 (0.00445)	0.757 (0.00674)	0.661 (0.00278)	0.711 (0.0141)	0.515 (0.00234)	0.673 (0.00413)	0.475 (0.00225)	0.667 (0.00594)

h superkingdom - CAMI II mouse gut dataset

Tool	Gold standard	CommonKmers	FOCUS	Metaphlan	MetaPhyler	Quikr	TIPP	mOTU
**Presence/absence of taxa**								
Completeness (recall)	1 (0)	1 (0)	1 (0)	1 (0)	1 (0)	1 (0)	1 (0)	1 (0)
Purity (precision)	1 (0)	0.922 (0.0229)	0.555 (0.0197)	1 (0)	0.5 (0)	0.333 (0)	0.5 (0)	1 (0)
F1 score	1 (0)	0.948 (0.0152)	0.703 (0.0131)	1 (0)	0.667 (0)	0.5 (0)	0.667 (0)	1 (0)
True positives	1 (0)	1 (0)	1 (0)	1 (0)	1 (0)	1 (0)	1 (0)	1 (0)
False positives	0 (0)	0.156 (0.0457)	0.891 (0.0393)	0 (0)	1 (0)	2 (0)	1 (0)	0 (0)
False negatives	0 (0)	0 (0)	0 (0)	0 (0)	0 (0)	0 (0)	0 (0)	0 (0)
Jaccard index	1 (0)	0.922 (0.0229)	0.555 (0.0197)	1 (0)	0.5 (0)	0.333 (0)	0.5 (0)	1 (0)
**Abundance estimates**								
Weighted UniFrac error	0 (0)	3.75 (0.184)	6.69 (0.364)	2.98 (0.124)	4.32 (0.0691)	7.27 (0.0765)	4.65 (0.078)	4.95 (0.196)
Unweighted UniFrac error	0 (0)	1.59e+03 (71.8)	2.47e+03 (98.2)	1.78e+03 (86)	7.86e+03 (154)	7.15e+03 (81.4)	5.36e+03 (88.9)	1.96e+03 (76.7)
L1 norm error	0 (0)	0.00459 (0.00309)	0.0644 (0.00389)	0 (0)	0.000428 (3.13e-05)	0.0925 (0.00217)	0.00581 (0.000197)	0 (0)
Bray-Curtis distance	0 (0)	0.00229 (0.00154)	0.0322 (0.00194)	0 (0)	0.000214 (1.56e-05)	0.0462 (0.00109)	0.0029 (9.87e-05)	0 (0)
**Alpha diversity**								
Taxon counts	1 (0)	1.16 (0.0457)	1.89 (0.0393)	1 (0)	2 (0)	3 (0)	2 (0)	1 (0)
Shannon diversity	0 (0)	0.00871 (0.00558)	0.137 (0.00753)	0 (0)	0.00198 (0.000134)	0.216 (0.00378)	0.0197 (0.000583)	0 (0)
Shannon equitability	0 (0)	0.0126 (0.00805)	0.197 (0.0109)	0 (0)	0.00286 (0.000193)	0.197 (0.00344)	0.0285 (0.000841)	0 (0)

i phylum - CAMI II mouse gut dataset

Tool	Gold standard	CommonKmers	FOCUS	Metaphlan	MetaPhyler	Quikr	TIPP	mOTU
**Presence/absence of taxa**								
Completeness (recall)	1 (0)	0.898 (0.0142)	0.828 (0.0299)	0.959 (0.0104)	0.985 (0.00634)	0.997 (0.00312)	0.985 (0.00634)	0.939 (0.0129)
Purity (precision)	1 (0)	0.932 (0.0138)	0.3 (0.00734)	1 (0)	0.228 (0.00396)	0.139 (0.00255)	0.189 (0.0034)	0.974 (0.00799)
F1 score	1 (0)	0.91 (0.0115)	0.424 (0.0107)	0.977 (0.00581)	0.369 (0.00522)	0.244 (0.00389)	0.316 (0.00475)	0.951 (0.00766)
True positives	4.67 (0.0773)	4.17 (0.0789)	3.83 (0.135)	4.47 (0.0802)	4.59 (0.0729)	4.66 (0.0779)	4.59 (0.0729)	4.38 (0.0848)
False positives	0 (0)	0.359 (0.0751)	9.3 (0.405)	0 (0)	15.7 (0.171)	28.9 (0.239)	19.8 (0.173)	0.141 (0.0438)
False negatives	0 (0)	0.5 (0.0704)	0.844 (0.141)	0.203 (0.0507)	0.0781 (0.0338)	0.0156 (0.0156)	0.0781 (0.0338)	0.297 (0.0617)
Jaccard index	1 (0)	0.847 (0.0188)	0.272 (0.00853)	0.959 (0.0104)	0.227 (0.00397)	0.139 (0.00256)	0.189 (0.00343)	0.913 (0.0134)
**Abundance estimates**								
Weighted UniFrac error	0 (0)	3.75 (0.184)	6.69 (0.364)	2.98 (0.124)	4.32 (0.0691)	7.27 (0.0765)	4.65 (0.078)	4.95 (0.196)
Unweighted UniFrac error	0 (0)	1.59e+03 (71.8)	2.47e+03 (98.2)	1.78e+03 (86)	7.86e+03 (154)	7.15e+03 (81.4)	5.36e+03 (88.9)	1.96e+03 (76.7)
L1 norm error	0 (0)	0.239 (0.027)	0.495 (0.065)	0.18 (0.0182)	0.0624 (0.00655)	0.557 (0.0179)	0.159 (0.0102)	0.273 (0.0358)
Bray-Curtis distance	0 (0)	0.12 (0.0135)	0.248 (0.0325)	0.0902 (0.00909)	0.0312 (0.00327)	0.278 (0.00896)	0.0794 (0.00509)	0.136 (0.0179)
**Alpha diversity**								
Taxon counts	4.67 (0.0773)	4.53 (0.102)	13.1 (0.504)	4.47 (0.0802)	20.2 (0.157)	33.5 (0.225)	24.4 (0.152)	4.52 (0.0996)
Shannon diversity	0.699 (0.0341)	0.706 (0.0319)	1.24 (0.0269)	0.655 (0.0352)	0.737 (0.0349)	1.69 (0.0175)	0.873 (0.0313)	0.637 (0.0362)
Shannon equitability	0.336 (0.0164)	0.478 (0.0222)	0.52 (0.0183)	0.444 (0.0236)	0.245 (0.0115)	0.48 (0.00456)	0.265 (0.0095)	0.43 (0.0238)

j class - CAMI II mouse gut dataset

Tool	Gold standard	CommonKmers	FOCUS	Metaphlan	MetaPhyler	Quikr	TIPP	mOTU
**Presence/absence of taxa**								
Completeness (recall)	1 (0)	0.661 (0.0119)	0.626 (0.0288)	0.813 (0.0104)	0.992 (0.00341)	0.761 (0.00899)	0.992 (0.00341)	0.818 (0.0113)
Purity (precision)	1 (0)	0.757 (0.0132)	0.285 (0.0134)	1 (0)	0.227 (0.00475)	0.155 (0.00333)	0.207 (0.00483)	0.916 (0.0115)
F1 score	1 (0)	0.698 (0.00872)	0.426 (0.00952)	0.895 (0.00632)	0.368 (0.00636)	0.257 (0.00478)	0.341 (0.00669)	0.858 (0.00704)
True positives	9.89 (0.215)	6.52 (0.17)	6.16 (0.31)	8 (0.173)	9.81 (0.216)	7.5 (0.174)	9.81 (0.216)	8.05 (0.179)
False positives	0 (0)	2.17 (0.142)	13.9 (0.559)	0 (0)	33.5 (0.375)	40.8 (0.371)	37.9 (0.454)	0.812 (0.113)
False negatives	0 (0)	3.38 (0.15)	3.73 (0.298)	1.89 (0.118)	0.0781 (0.0338)	2.39 (0.106)	0.0781 (0.0338)	1.84 (0.126)
Jaccard index	1 (0)	0.541 (0.0104)	0.248 (0.0121)	0.813 (0.0104)	0.227 (0.00476)	0.148 (0.00318)	0.206 (0.00485)	0.755 (0.0111)
**Abundance estimates**								
Weighted UniFrac error	0 (0)	3.75 (0.184)	6.69 (0.364)	2.98 (0.124)	4.32 (0.0691)	7.27 (0.0765)	4.65 (0.078)	4.95 (0.196)
Unweighted UniFrac error	0 (0)	1.59e+03 (71.8)	2.47e+03 (98.2)	1.78e+03 (86)	7.86e+03 (154)	7.15e+03 (81.4)	5.36e+03 (88.9)	1.96e+03 (76.7)
L1 norm error	0 (0)	0.323 (0.0261)	0.584 (0.0627)	0.215 (0.0178)	0.0984 (0.008)	0.66 (0.0148)	0.217 (0.0128)	0.369 (0.0351)
Bray-Curtis distance	0 (0)	0.161 (0.0131)	0.292 (0.0313)	0.107 (0.00892)	0.0492 (0.004)	0.33 (0.00738)	0.108 (0.00641)	0.184 (0.0175)
**Alpha diversity**								
Taxon counts	9.89 (0.215)	8.69 (0.216)	20 (0.804)	8 (0.173)	43.3 (0.385)	48.3 (0.4)	47.7 (0.398)	8.86 (0.212)
Shannon diversity	1 (0.0337)	0.962 (0.0341)	1.63 (0.041)	0.897 (0.0346)	1.09 (0.0376)	2.17 (0.0235)	1.25 (0.0352)	0.888 (0.0407)
Shannon equitability	0.347 (0.0116)	0.449 (0.0159)	0.587 (0.0163)	0.437 (0.0177)	0.29 (0.00972)	0.56 (0.00558)	0.312 (0.00874)	0.41 (0.0184)

Fig. S6. Assessment summary of the results of profilers on the CAMI I high complexity (a-g) and the CAMI II mouse gut (h-n) datasets. The values of the metrics are averaged over all samples in each dataset, with the standard error being shown in parentheses.

Worst Median Best

d order - CAMI I high complexity dataset

Presence/absence of taxa								
Tool	Gold standard	CommonKmers	FOCUS	Metaphlan	MetaPhyler	Quikr	TIPP	mOTU
Completeness (recall)	1 (0)	0.535 (0.017)	0.78 (0.0215)	0.675 (0.0112)	0.975 (0)	1 (0)	0.995 (0.005)	0.72 (0.0122)
Purity (precision)	1 (0)	0.983 (0.0104)	0.547 (0.00775)	0.763 (0.00607)	0.323 (0.00182)	0.355 (0.00412)	0.369 (0.00171)	0.566 (0.0129)
F1 score	1 (0)	0.692 (0.013)	0.643 (0.0126)	0.716 (0.00796)	0.486 (0.00205)	0.524 (0.0045)	0.538 (0.00203)	0.633 (0.0106)
True positives	40 (0)	21.4 (0.678)	31.2 (0.86)	27 (0.447)	39 (0)	40 (0)	39.8 (0.2)	28.8 (0.49)
False positives	0 (0)	0.4 (0.245)	25.8 (0.2)	8.4 (0.245)	81.6 (0.678)	72.8 (1.32)	68.2 (0.583)	22.2 (1.11)
False negatives	0 (0)	18.6 (0.678)	8.8 (0.86)	13 (0.447)	1 (0)	0 (0)	0.2 (0.2)	11.2 (0.49)
Jaccard index	1 (0)	0.53 (0.0149)	0.474 (0.0135)	0.558 (0.00964)	0.321 (0.00179)	0.355 (0.00412)	0.368 (0.0019)	0.464 (0.0114)
**Abundance estimates**								
Weighted UniFrac error	0 (0)	7.65 (0.0572)	7.79 (0.0963)	9.49 (0.0828)	6.45 (0.0196)	7.81 (0.0582)	7 (0.0352)	8.74 (0.0447)
Unweighted UniFrac error	0 (0)	5.56e+03 (97.8)	5.92e+03 (79.2)	6.27e+03 (39.1)	1.22e+04 (68.3)	1e+04 (148)	7.46e+03 (49.6)	6.48e+03 (68.7)
L1 norm error	0 (0)	0.567 (0.00983)	0.47 (0.0256)	1.13 (0.0227)	0.681 (0.00187)	0.648 (0.0178)	0.818 (0.0043)	1.08 (0.0194)
Bray-Curtis distance	0 (0)	0.284 (0.00492)	0.235 (0.0128)	0.563 (0.0113)	0.34 (0.000935)	0.324 (0.0089)	0.409 (0.00215)	0.538 (0.00971)
**Alpha diversity**								
Taxon counts	40 (0)	21.8 (0.86)	57 (0.837)	35.4 (0.51)	121 (0.678)	113 (1.32)	108 (0.707)	51 (1.22)
Shannon diversity	2.76 (0.0147)	2.24 (0.0276)	2.9 (0.0287)	2.63 (0.0379)	3.13 (0.0114)	3.54 (0.00904)	3.04 (0.00761)	2.58 (0.0143)
Shannon equitability	0.747 (0.00399)	0.728 (0.00297)	0.717 (0.00569)	0.739 (0.0129)	0.652 (0.00193)	0.75 (0.00344)	0.626 (0.00158)	0.657 (0.0054)

e family - CAMI I high complexity dataset

Presence/absence of taxa								
Tool	Gold standard	CommonKmers	FOCUS	Metaphlan	MetaPhyler	Quikr	TIPP	mOTU
Completeness (recall)	1 (0)	0.452 (0.0127)	0.65 (0.0121)	0.573 (0.00455)	0.92 (0)	0.916 (0.00278)	0.923 (0.00663)	0.677 (0.0085)
Purity (precision)	1 (0)	0.966 (0.00986)	0.536 (0.00684)	0.849 (0.00738)	0.322 (0.00104)	0.367 (0.00547)	0.389 (0.0035)	0.584 (0.00849)
F1 score	1 (0)	0.616 (0.0131)	0.587 (0.00866)	0.684 (0.0026)	0.477 (0.00114)	0.524 (0.00586)	0.548 (0.00401)	0.627 (0.00834)
True positives	88 (0)	39.8 (1.11)	57.2 (1.07)	50.4 (0.4)	81 (0)	80.6 (0.245)	81.2 (0.583)	59.6 (0.748)
False positives	0 (0)	1.4 (0.4)	49.6 (0.812)	9 (0.548)	170 (0.812)	139 (3.08)	127 (1.83)	42.4 (1.03)
False negatives	0 (0)	48.2 (1.11)	30.8 (1.07)	37.6 (0.4)	7 (0)	7.4 (0.245)	6.8 (0.583)	28.4 (0.748)
Jaccard index	1 (0)	0.445 (0.0136)	0.416 (0.00879)	0.52 (0.003)	0.313 (0.000988)	0.355 (0.00537)	0.377 (0.0038)	0.457 (0.00876)
**Abundance estimates**								
Weighted UniFrac error	0 (0)	7.65 (0.0572)	7.79 (0.0963)	9.49 (0.0828)	6.45 (0.0196)	7.81 (0.0582)	7 (0.0352)	8.74 (0.0447)
Unweighted UniFrac error	0 (0)	5.56e+03 (97.8)	5.92e+03 (79.2)	6.27e+03 (39.1)	1.22e+04 (68.3)	1e+04 (148)	7.46e+03 (49.6)	6.48e+03 (68.7)
L1 norm error	0 (0)	0.923 (0.0222)	0.787 (0.0172)	1.24 (0.0155)	0.442 (0.00771)	0.975 (0.0168)	0.508 (0.0113)	1.01 (0.0112)
Bray-Curtis distance	0 (0)	0.461 (0.0111)	0.393 (0.00858)	0.62 (0.00775)	0.221 (0.00386)	0.487 (0.00842)	0.254 (0.00567)	0.505 (0.0056)
**Alpha diversity**								
Taxon counts	88 (0)	41.2 (0.97)	107 (1.16)	59.4 (0.872)	251 (0.812)	220 (2.97)	209 (2.01)	102 (0.632)
Shannon diversity	3.74 (0.00852)	3.06 (0.0231)	4.05 (0.0197)	2.92 (0.0576)	3.97 (0.0127)	4.7 (0.00168)	3.9 (0.0146)	3.15 (0.00654)
Shannon equitability	0.835 (0.0019)	0.824 (0.00373)	0.868 (0.00349)	0.715 (0.0164)	0.717 (0.00196)	0.871 (0.00239)	0.697 (0.00269)	0.681 (0.00122)

f genus - CAMI I high complexity dataset

Presence/absence of taxa								
Tool	Gold standard	CommonKmers	FOCUS	Metaphlan	MetaPhyler	Quikr	TIPP	mOTU
Completeness (recall)	1 (0)	0.269 (0.00412)	0.246 (0.00526)	0.314 (0)	0.432 (0.00103)	0.593 (0.0118)	0.431 (0.00126)	0.336 (0.0064)
Purity (precision)	1 (0)	0.772 (0.00902)	0.265 (0.00768)	0.761 (0.00934)	0.146 (0.000378)	0.186 (0.00433)	0.193 (0.000963)	0.306 (0.00499)
F1 score	1 (0)	0.399 (0.00532)	0.255 (0.00582)	0.445 (0.00158)	0.218 (0.000418)	0.283 (0.0062)	0.267 (0.001)	0.32 (0.00552)
True positives	194 (0)	52.2 (0.8)	47.8 (1.02)	61 (0)	83.8 (0.2)	115 (2.28)	83.6 (0.245)	65.2 (1.24)
False positives	0 (0)	15.4 (0.678)	133 (4.23)	19.2 (0.97)	490 (2.06)	504 (8.07)	350 (2.18)	148 (1.56)
False negatives	0 (0)	142 (0.8)	146 (1.02)	133 (0)	110 (0.2)	79 (2.28)	110 (0.245)	129 (1.24)
Jaccard index	1 (0)	0.249 (0.00415)	0.146 (0.00383)	0.286 (0.00131)	0.123 (0.000263)	0.165 (0.00418)	0.154 (0.000666)	0.191 (0.00391)
**Abundance estimates**								
Weighted UniFrac error	0 (0)	7.65 (0.0572)	7.79 (0.0963)	9.49 (0.0828)	6.45 (0.0196)	7.81 (0.0582)	7 (0.0352)	8.74 (0.0447)
Unweighted UniFrac error	0 (0)	5.56e+03 (97.8)	5.92e+03 (79.2)	6.27e+03 (39.1)	1.22e+04 (68.3)	1e+04 (148)	7.46e+03 (49.6)	6.48e+03 (68.7)
L1 norm error	0 (0)	1.19 (0.0145)	1.34 (0.0203)	1.5 (0.0108)	1.13 (0.0107)	1.5 (0.00839)	1.2 (0.012)	1.4 (0.00979)
Bray-Curtis distance	0 (0)	0.597 (0.00726)	0.669 (0.0101)	0.752 (0.00539)	0.564 (0.00534)	0.748 (0.0042)	0.599 (0.00602)	0.698 (0.00489)
**Alpha diversity**								
Taxon counts	194 (0)	67.6 (0.872)	181 (4.24)	80.2 (0.97)	574 (2.2)	619 (7.69)	433 (2.25)	213 (1.79)
Shannon diversity	4.54 (0.0151)	3.42 (0.0238)	4.61 (0.0258)	3.09 (0.0632)	4.84 (0.0176)	5.74 (0.00678)	4.78 (0.0127)	3.57 (0.0198)
Shannon equitability	0.862 (0.00286)	0.813 (0.0043)	0.887 (0.00291)	0.706 (0.0154)	0.762 (0.0025)	0.892 (0.00178)	0.748 (0.00189)	0.666 (0.00295)

k order - CAMI II mouse gut dataset

Presence/absence of taxa								
Tool	Gold standard	CommonKmers	FOCUS	Metaphlan	MetaPhyler	Quikr	TIPP	mOTU
Completeness (recall)	1 (0)	0.667 (0.0115)	0.562 (0.0277)	0.748 (0.011)	0.97 (0.00616)	0.791 (0.0102)	0.967 (0.00632)	0.749 (0.0136)
Purity (precision)	1 (0)	0.773 (0.0193)	0.184 (0.00911)	1 (0)	0.115 (0.00286)	0.103 (0.00306)	0.112 (0.00311)	0.825 (0.0177)
F1 score	1 (0)	0.705 (0.011)	0.3 (0.00819)	0.853 (0.00729)	0.204 (0.0046)	0.182 (0.0049)	0.2 (0.00502)	0.771 (0.00945)
True positives	12 (0.325)	7.97 (0.256)	6.7 (0.373)	8.81 (0.199)	11.6 (0.314)	9.47 (0.295)	11.5 (0.317)	8.83 (0.223)
False positives	0 (0)	2.52 (0.237)	26.8 (1.11)	0 (0)	89.3 (0.845)	82 (0.65)	92 (0.988)	2.27 (0.282)
False negatives	0 (0)	3.98 (0.175)	5.25 (0.343)	3.14 (0.194)	0.375 (0.0755)	2.48 (0.12)	0.406 (0.0762)	3.12 (0.214)
Jaccard index	1 (0)	0.551 (0.0127)	0.161 (0.0083)	0.748 (0.011)	0.114 (0.00285)	0.101 (0.00298)	0.112 (0.0031)	0.634 (0.0129)
**Abundance estimates**								
Weighted UniFrac error	0 (0)	3.75 (0.184)	6.69 (0.364)	2.98 (0.124)	4.32 (0.0691)	7.27 (0.0765)	4.65 (0.078)	4.95 (0.196)
Unweighted UniFrac error	0 (0)	1.59e+03 (71.8)	2.47e+03 (98.2)	1.78e+03 (86)	7.86e+03 (154)	7.15e+03 (81.4)	5.36e+03 (88.9)	1.96e+03 (76.7)
L1 norm error	0 (0)	0.318 (0.0258)	0.646 (0.06)	0.22 (0.0179)	0.112 (0.00816)	0.773 (0.0143)	0.227 (0.0128)	0.374 (0.0348)
Bray-Curtis distance	0 (0)	0.159 (0.0129)	0.323 (0.03)	0.11 (0.00895)	0.056 (0.00408)	0.387 (0.00715)	0.113 (0.0064)	0.187 (0.0174)
**Alpha diversity**								
Taxon counts	12 (0.325)	10.5 (0.305)	33.5 (1.41)	8.81 (0.199)	101 (0.916)	91.5 (0.682)	104 (0.95)	11.1 (0.404)
Shannon diversity	1.02 (0.0339)	0.976 (0.0342)	1.84 (0.0436)	0.901 (0.034)	1.18 (0.0389)	2.54 (0.0275)	1.37 (0.0354)	0.894 (0.0399)
Shannon equitability	0.313 (0.0104)	0.42 (0.0145)	0.568 (0.0156)	0.42 (0.0163)	0.256 (0.00821)	0.562 (0.00555)	0.282 (0.00727)	0.38 (0.0169)

l family - CAMI II mouse gut dataset

Presence/absence of taxa								
Tool	Gold standard	CommonKmers	FOCUS	Metaphlan	MetaPhyler	Quikr	TIPP	mOTU
Completeness (recall)	1 (0)	0.618 (0.0104)	0.476 (0.0233)	0.742 (0.009)	0.875 (0.00854)	0.694 (0.00766)	0.867 (0.00891)	0.712 (0.0105)
Purity (precision)	1 (0)	0.783 (0.0214)	0.203 (0.00956)	0.963 (0.00522)	0.099 (0.00192)	0.0997 (0.00239)	0.107 (0.00247)	0.774 (0.0165)
F1 score	1 (0)	0.677 (0.0105)	0.31 (0.00757)	0.836 (0.00644)	0.177 (0.00308)	0.174 (0.00367)	0.19 (0.0039)	0.732 (0.00895)
True positives	23.4 (0.621)	14.4 (0.392)	11.1 (0.598)	17.2 (0.399)	20.2 (0.448)	16.1 (0.402)	20 (0.44)	16.4 (0.337)
False positives	0 (0)	4.42 (0.513)	38.5 (1.67)	0.703 (0.0988)	185 (2.3)	146 (1.19)	168 (2.15)	5.41 (0.545)
False negatives	0 (0)	9.02 (0.362)	12.3 (0.645)	6.19 (0.312)	3.12 (0.256)	7.25 (0.296)	3.33 (0.274)	6.98 (0.382)
Jaccard index	1 (0)	0.517 (0.0115)	0.167 (0.00824)	0.722 (0.00961)	0.0975 (0.00186)	0.0953 (0.0022)	0.105 (0.00238)	0.582 (0.0113)
**Abundance estimates**								
Weighted UniFrac error	0 (0)	3.75 (0.184)	6.69 (0.364)	2.98 (0.124)	4.32 (0.0691)	7.27 (0.0765)	4.65 (0.078)	4.95 (0.196)
Unweighted UniFrac error	0 (0)	1.59e+03 (71.8)	2.47e+03 (98.2)	1.78e+03 (86)	7.86e+03 (154)	7.15e+03 (81.4)	5.36e+03 (88.9)	1.96e+03 (76.7)
L1 norm error	0 (0)	0.536 (0.0445)	0.863 (0.0587)	0.273 (0.0172)	0.267 (0.0133)	1.07 (0.0182)	0.329 (0.0155)	0.482 (0.0372)
Bray-Curtis distance	0 (0)	0.268 (0.0223)	0.431 (0.0293)	0.137 (0.00861)	0.133 (0.00664)	0.535 (0.00908)	0.165 (0.00773)	0.241 (0.0186)
**Alpha diversity**								
Taxon counts	23.4 (0.621)	18.8 (0.504)	49.5 (2.19)	17.9 (0.43)	205 (2.5)	162 (1.24)	188 (2.18)	21.8 (0.674)
Shannon diversity	1.51 (0.0575)	1.45 (0.0522)	2.33 (0.0711)	1.36 (0.0572)	1.85 (0.0576)	3.39 (0.0356)	1.92 (0.0589)	1.35 (0.0668)
Shannon equitability	0.387 (0.0147)	0.496 (0.0177)	0.633 (0.0145)	0.47 (0.0183)	0.346 (0.0104)	0.667 (0.00635)	0.346 (0.0106)	0.439 (0.0211)

m genus - CAMI II mouse gut dataset

Presence/absence of taxa								
Tool	Gold standard	CommonKmers	FOCUS	Metaphlan	MetaPhyler	Quikr	TIPP	mOTU
Completeness (recall)	1 (0)	0.537 (0.0123)	0.247 (0.0138)	0.582 (0.00836)	0.539 (0.0108)	0.487 (0.0123)	0.528 (0.0109)	0.509 (0.00885)
Purity (precision)	1 (0)	0.772 (0.0234)	0.163 (0.00873)	0.944 (0.00543)	0.0654 (0.00186)	0.0672 (0.00242)	0.0846 (0.00241)	0.689 (0.0167)
F1 score	1 (0)	0.635 (0.0123)	0.212 (0.00729)	0.718 (0.00637)	0.116 (0.00292)	0.117 (0.00389)	0.144 (0.00356)	0.574 (0.00665)
True positives	51.9 (1.95)	29 (1.07)	12.8 (0.841)	29.5 (0.886)	26.9 (0.697)	25.1 (1.04)	26.4 (0.689)	25.6 (0.735)
False positives	0 (0)	9.02 (1.06)	56.9 (2.64)	1.7 (0.151)	389 (6.88)	343 (3.76)	288 (4.06)	12.2 (0.896)
False negatives	0 (0)	22.9 (1.13)	39.2 (1.62)	22.5 (1.12)	25 (1.35)	26.9 (1.21)	25.6 (1.36)	26.3 (1.29)
Jaccard index	1 (0)	0.473 (0.0123)	0.108 (0.00599)	0.562 (0.00806)	0.0616 (0.00166)	0.0625 (0.00219)	0.078 (0.00205)	0.405 (0.00673)
**Abundance estimates**								
Weighted UniFrac error	0 (0)	3.75 (0.184)	6.69 (0.364)	2.98 (0.124)	4.32 (0.0691)	7.27 (0.0765)	4.65 (0.078)	4.95 (0.196)
Unweighted UniFrac error	0 (0)	1.59e+03 (71.8)	2.47e+03 (98.2)	1.78e+03 (86)	7.86e+03 (154)	7.15e+03 (81.4)	5.36e+03 (88.9)	1.96e+03 (76.7)
L1 norm error	0 (0)	0.638 (0.0477)	0.964 (0.055)	0.332 (0.0207)	0.514 (0.0229)	1.2 (0.0198)	0.497 (0.0235)	0.592 (0.0434)
Bray-Curtis distance	0 (0)	0.319 (0.0239)	0.482 (0.0275)	0.166 (0.0103)	0.257 (0.0114)	0.598 (0.0099)	0.249 (0.0118)	0.296 (0.0217)
**Alpha diversity**								
Taxon counts	51.9 (1.95)	38 (0.983)	69.6 (3.35)	31.2 (0.911)	416 (7.12)	368 (4.38)	315 (4.07)	37.8 (1.14)
Shannon diversity	1.88 (0.0864)	1.85 (0.0777)	2.63 (0.0918)	1.6 (0.0769)	2.41 (0.0812)	4.09 (0.0518)	2.38 (0.0847)	1.5 (0.0844)
Shannon equitability	0.372 (0.0171)	0.507 (0.0201)	0.658 (0.0172)	0.463 (0.0202)	0.399 (0.0129)	0.693 (0.00769)	0.379 (0.0134)	0.412 (0.0222)

Fig. S7. Assessment summary of the results of profilers on the CAMI I high complexity (a-g) and the CAMI II mouse gut (h-n) datasets. The values of the metrics are averaged over all samples in each dataset, with the standard error being shown in parentheses.

Worst Median Best

g species - CAMI I high complexity dataset

Presence/absence of taxa

Tool	Gold standard	CommonKmers	FOCUS	Metaphlan	MetaPhyler	Quikr	TIPP	mOTU
Completeness (recall)	1 (0)	0.073 (0.00212)	0.00166 (0.00102)	0.0158 (0.00083)	0.0166 (0)	0.146 (0.00578)	0.0166 (0)	0.0108 (0.00102)
Purity (precision)	1 (0)	0.0811 (0.00183)	0.00151 (0.000923)	0.0734 (0.004)	0.00301 (9.63e-06)	0.0326 (0.00134)	0.00521 (2.22e-05)	0.00605 (0.000553)
F1 score	1 (0)	0.0768 (0.00194)	0.00395 (4.29e-05)	0.026 (0.00136)	0.00509 (1.38e-05)	0.0533 (0.00216)	0.00794 (2.57e-05)	0.00775 (0.000714)
True positives	241 (0)	17.6 (0.51)	0.4 (0.245)	3.8 (0.2)	4 (0)	35.2 (1.39)	4 (0)	2.6 (0.245)
False positives	0 (0)	199 (2.38)	269 (4.26)	48 (1.05)	1.33e+03 (4.25)	1.04e+03 (13.7)	763 (3.26)	427 (5.72)
False negatives	0 (0)	223 (0.51)	241 (0.245)	237 (0.2)	237 (0)	206 (1.39)	237 (0)	238 (0.245)
Jaccard index	1 (0)	0.04 (0.00105)	0.000791 (0.000485)	0.0131 (0.000694)	0.00255 (6.93e-06)	0.0274 (0.00114)	0.00398 (1.29e-05)	0.00389 (0.00036)

Abundance estimates

Weighted UniFrac error	0 (0)	7.65 (0.0572)	7.79 (0.0963)	9.49 (0.0828)	6.45 (0.0196)	7.81 (0.0582)	7 (0.0352)	8.74 (0.0447)
Unweighted UniFrac error	0 (0)	5.56e+03 (97.8)	5.92e+03 (79.2)	6.27e+03 (39.1)	1.22e+04 (68.3)	1e+04 (148)	7.46e+03 (49.6)	6.48e+03 (68.7)
L1 norm error	0 (0)	1.79 (0.00955)	2 (0.00063)	1.89 (0.00575)	1.94 (0.00201)	1.94 (0.00103)	1.93 (0.00294)	1.95 (0.00525)
Bray-Curtis distance	0 (0)	0.896 (0.00478)	0.999 (0.000315)	0.947 (0.00287)	0.97 (0.00101)	0.97 (0.000514)	0.964 (0.00147)	0.974 (0.00263)

Alpha diversity

Taxon counts	241 (0)	217 (2.7)	270 (4.15)	51.8 (1.07)	1.33e+03 (4.25)	1.08e+03 (13.7)	767 (3.26)	430 (5.79)
Shannon diversity	5.06 (0.0355)	4.72 (0.0492)	5.11 (0.0152)	2.49 (0.0736)	5.65 (0.0187)	6.36 (0.00352)	5.38 (0.0152)	4.24 (0.0347)
Shannon equitability	0.922 (0.00647)	0.877 (0.00842)	0.913 (0.00233)	0.631 (0.0186)	0.786 (0.00231)	0.911 (0.00118)	0.751 (0.00201)	0.699 (0.00544)

n species - CAMI II mouse gut dataset

Presence/absence of taxa

Tool	Gold standard	CommonKmers	FOCUS	Metaphlan	MetaPhyler	Quikr	TIPP	mOTU
Completeness (recall)	1 (0)	0.467 (0.0132)	0.128 (0.00801)	0.452 (0.0062)	0.303 (0.0059)	0.257 (0.00602)	0.295 (0.00566)	0.339 (0.00577)
Purity (precision)	1 (0)	0.552 (0.0199)	0.138 (0.0073)	0.891 (0.00416)	0.0505 (0.00175)	0.0547 (0.00262)	0.081 (0.0026)	0.466 (0.0101)
F1 score	1 (0)	0.484 (0.0109)	0.141 (0.00457)	0.598 (0.00558)	0.0852 (0.0025)	0.0886 (0.00374)	0.124 (0.00306)	0.386 (0.00427)
True positives	149 (6.86)	68.4 (3.36)	18.3 (1.25)	65.4 (2.66)	43.1 (1.56)	38.4 (1.97)	42 (1.56)	48.6 (1.86)
False positives	0 (0)	56.9 (3.32)	99.2 (4.87)	7.92 (0.384)	816 (15.7)	655 (8.72)	474 (6.12)	54.8 (1.7)
False negatives	0 (0)	80.5 (4.2)	131 (6.2)	83.5 (4.32)	106 (5.39)	110 (5.12)	107 (5.39)	100 (5.09)
Jaccard index	1 (0)	0.324 (0.00914)	0.0689 (0.00365)	0.428 (0.00573)	0.0446 (0.00136)	0.0466 (0.00205)	0.0665 (0.00173)	0.239 (0.00324)

Abundance estimates

Weighted UniFrac error	0 (0)	3.75 (0.184)	6.69 (0.364)	2.98 (0.124)	4.32 (0.0691)	7.27 (0.0765)	4.65 (0.078)	4.95 (0.196)
Unweighted UniFrac error	0 (0)	1.59e+03 (71.8)	2.47e+03 (98.2)	1.78e+03 (86)	7.86e+03 (154)	7.15e+03 (81.4)	5.36e+03 (88.9)	1.96e+03 (76.7)
L1 norm error	0 (0)	0.819 (0.0318)	1.4 (0.0385)	0.845 (0.0387)	1.26 (0.0399)	1.68 (0.0162)	1.21 (0.0425)	1.3 (0.0317)
Bray-Curtis distance	0 (0)	0.409 (0.0159)	0.699 (0.0192)	0.422 (0.0194)	0.632 (0.0199)	0.84 (0.00809)	0.605 (0.0212)	0.648 (0.0159)

Alpha diversity

Taxon counts	149 (6.86)	125 (4.43)	117 (5.95)	73.3 (2.93)	860 (16.3)	693 (9.5)	516 (6.87)	103 (2.99)
Shannon diversity	2.96 (0.0928)	3.09 (0.101)	3.59 (0.114)	2.39 (0.0904)	3.82 (0.078)	5.52 (0.0402)	3.56 (0.0794)	2.46 (0.0823)
Shannon equitability	0.469 (0.0147)	0.64 (0.0178)	0.795 (0.0109)	0.561 (0.019)	0.565 (0.0105)	0.845 (0.00492)	0.511 (0.0113)	0.529 (0.0157)

Fig. S8. Assessment summary of the results of profilers on the CAMI I high complexity (a-g) and the CAMI II mouse gut (h-n) datasets. The values of the metrics are averaged over all samples in each dataset, with the standard error being shown in parentheses.

Worst Median Best

a superkingdom - HMP Mock Community dataset

Tool	Gold standard	CommonKmers	FOCUS	Metaphlan	MetaPhyler	Quikr	mOTU
**Presence/absence of taxa**							
Completeness (recall)	1	1	0.667	0.667	0.667	1	0.667
Purity (precision)	1	1	1	1	1	1	1
F1 score	1	1	0.8	0.8	0.8	1	0.8
True positives	3	3	2	2	2	3	2
False positives	0	0	0	0	0	0	0
False negatives	0	0	1	1	1	0	1
Jaccard index	1	1	0.667	0.667	0.667	1	0.667
**Abundance estimates**							
Weighted UniFrac error	0	8.11	8.98	5.83	5.99	11.3	6.45
Unweighted UniFrac error	0	213	475	142	1.76e+03	7.46e+03	3.22e+03
L1 norm error	0	0.206	0.324	0.241	0.24	0.134	0.171
Bray-Curtis distance	0	0.103	0.162	0.12	0.12	0.0671	0.0854
**Alpha diversity**							
Taxon counts	3	3	2	2	2	3	2
Shannon diversity	0.473	0.343	0.0898	0.226	0.226	0.546	0.313
Shannon equitability	0.43	0.312	0.129	0.326	0.326	0.497	0.451

b phylum - HMP Mock Community dataset

Tool	Gold standard	CommonKmers	FOCUS	Metaphlan	MetaPhyler	Quikr	mOTU
**Presence/absence of taxa**							
Completeness (recall)	1	0.714	0.714	0.857	0.857	0.857	0.857
Purity (precision)	1	0.833	0.556	1	0.75	0.176	1
F1 score	1	0.769	0.625	0.923	0.8	0.293	0.923
True positives	7	5	5	6	6	6	6
False positives	0	1	4	0	2	28	0
False negatives	0	2	2	1	1	1	1
Jaccard index	1	0.625	0.455	0.857	0.667	0.171	0.857
**Abundance estimates**							
Weighted UniFrac error	0	8.11	8.98	5.83	5.99	11.3	6.45
Unweighted UniFrac error	0	213	475	142	1.76e+03	7.46e+03	3.22e+03
L1 norm error	0	0.999	0.508	0.448	0.46	1.03	0.784
Bray-Curtis distance	0	0.499	0.254	0.224	0.23	0.513	0.392
**Alpha diversity**							
Taxon counts	7	6	9	6	8	34	6
Shannon diversity	1.06	0.375	1.39	0.837	0.832	2.56	0.589
Shannon equitability	0.542	0.209	0.635	0.467	0.4	0.726	0.329

c class - HMP Mock Community dataset

Tool	Gold standard	CommonKmers	FOCUS	Metaphlan	MetaPhyler	Quikr	mOTU
**Presence/absence of taxa**							
Completeness (recall)	1	0.727	0.727	0.909	0.909	0.909	0.909
Purity (precision)	1	0.889	0.533	1	0.625	0.204	1
F1 score	1	0.8	0.615	0.952	0.741	0.333	0.952
True positives	11	8	8	10	10	10	10
False positives	0	1	7	0	6	39	0
False negatives	0	3	3	1	1	1	1
Jaccard index	1	0.667	0.444	0.909	0.588	0.2	0.909
**Abundance estimates**							
Weighted UniFrac error	0	8.11	8.98	5.83	5.99	11.3	6.45
Unweighted UniFrac error	0	213	475	142	1.76e+03	7.46e+03	3.22e+03
L1 norm error	0	0.916	0.863	0.51	0.496	1.44	0.804
Bray-Curtis distance	0	0.458	0.432	0.255	0.248	0.719	0.402
**Alpha diversity**							
Taxon counts	11	9	15	10	16	49	10
Shannon diversity	1.41	0.974	1.96	1.06	1.07	3	0.7
Shannon equitability	0.59	0.443	0.725	0.461	0.386	0.771	0.304

d order - HMP Mock Community dataset

Tool	Gold standard	CommonKmers	FOCUS	Metaphlan	MetaPhyler	Quikr	mOTU
**Presence/absence of taxa**							
Completeness (recall)	1	0.769	0.615	0.923	0.923	0.923	0.846
Purity (precision)	1	0.667	0.4	0.8	0.353	0.125	0.846
F1 score	1	0.714	0.485	0.857	0.511	0.22	0.846
True positives	13	10	8	12	12	12	11
False positives	0	5	12	3	22	84	2
False negatives	0	3	5	1	1	1	2
Jaccard index	1	0.556	0.32	0.75	0.343	0.124	0.733
**Abundance estimates**							
Weighted UniFrac error	0	8.11	8.98	5.83	5.99	11.3	6.45
Unweighted UniFrac error	0	213	475	142	1.76e+03	7.46e+03	3.22e+03
L1 norm error	0	1.07	1.18	0.622	0.641	1.61	0.866
Bray-Curtis distance	0	0.535	0.592	0.311	0.32	0.803	0.433
**Alpha diversity**							
Taxon counts	13	15	20	15	34	96	13
Shannon diversity	1.77	1.35	2.07	1.46	1.46	3.55	1.14
Shannon equitability	0.689	0.497	0.692	0.537	0.413	0.777	0.444

Fig. S9. Assessment summary of the results of profilers on the HMP Mock Community dataset. This dataset consists of one sample.

Worst Median Best

e family - HMP Mock Community dataset

Presence/absence of taxa

Tool	Gold standard	CommonKmers	FOCUS	Metaphlan	MetaPhyler	Quikr	mOTU
Completeness (recall)	1	0.579	0.263	0.842	0.947	0.947	0.842
Purity (precision)	1	0.55	0.238	0.889	0.353	0.101	0.941
F1 score	1	0.564	0.25	0.865	0.514	0.183	0.889
True positives	19	11	5	16	18	18	16
False positives	0	9	16	2	33	160	1
False negatives	0	8	14	3	1	1	3
Jaccard index	1	0.393	0.143	0.762	0.346	0.101	0.8

Abundance estimates

Weighted UniFrac error	0	8.11	8.98	5.83	5.99	11.3	6.45
Unweighted UniFrac error	0	213	475	142	1.76e+03	7.46e+03	3.22e+03
L1 norm error	0	1.13	1.25	0.657	0.678	1.72	0.902
Bray-Curtis distance	0	0.563	0.624	0.328	0.339	0.861	0.451

Alpha diversity

Taxon counts	19	20	21	18	51	178	17
Shannon diversity	1.86	1.37	2.3	1.47	1.5	4.13	1.17
Shannon equitability	0.631	0.459	0.757	0.51	0.382	0.797	0.412

f genus - HMP Mock Community dataset

Presence/absence of taxa

Tool	Gold standard	CommonKmers	FOCUS	Metaphlan	MetaPhyler	Quikr	mOTU
Completeness (recall)	1	0.579	0.211	0.789	0.947	0.895	0.842
Purity (precision)	1	0.55	0.16	0.833	0.205	0.0459	0.667
F1 score	1	0.564	0.182	0.811	0.336	0.0874	0.744
True positives	19	11	4	15	18	17	16
False positives	0	9	21	3	70	353	8
False negatives	0	8	15	4	1	2	3
Jaccard index	1	0.393	0.1	0.682	0.202	0.0457	0.593

Abundance estimates

Weighted UniFrac error	0	8.11	8.98	5.83	5.99	11.3	6.45
Unweighted UniFrac error	0	213	475	142	1.76e+03	7.46e+03	3.22e+03
L1 norm error	0	1.13	1.31	0.743	0.713	1.83	0.951
Bray-Curtis distance	0	0.563	0.657	0.371	0.357	0.913	0.475

Alpha diversity

Taxon counts	19	20	25	18	88	370	24
Shannon diversity	1.86	1.37	2.43	1.33	1.59	4.69	1.19
Shannon equitability	0.631	0.459	0.753	0.458	0.356	0.792	0.375

g species - HMP Mock Community dataset

Presence/absence of taxa

Tool	Gold standard	CommonKmers	FOCUS	Metaphlan	MetaPhyler	Quikr	mOTU
Completeness (recall)	1	0.409	0.136	0.591	0.955	0.227	0.818
Purity (precision)	1	0.321	0.0938	0.867	0.127	0.00803	0.375
F1 score	1	0.36	0.111	0.703	0.225	0.0155	0.514
True positives	22	9	3	13	21	5	18
False positives	0	19	29	2	144	618	30
False negatives	0	13	19	9	1	17	4
Jaccard index	1	0.22	0.0588	0.542	0.127	0.00781	0.346

Abundance estimates

Weighted UniFrac error	0	8.11	8.98	5.83	5.99	11.3	6.45
Unweighted UniFrac error	0	213	475	142	1.76e+03	7.46e+03	3.22e+03
L1 norm error	0	1.39	1.71	0.656	0.767	1.97	0.953
Bray-Curtis distance	0	0.694	0.854	0.328	0.384	0.986	0.476

Alpha diversity

Taxon counts	22	28	32	15	165	623	48
Shannon diversity	1.98	2.11	2.76	1.52	2.19	5.45	1.68
Shannon equitability	0.641	0.634	0.795	0.561	0.429	0.846	0.432

Fig. S10. Assessment summary of the results of profilers on the HMP Mock Community dataset. This dataset consists of one sample.

a CAMI I high complexity dataset

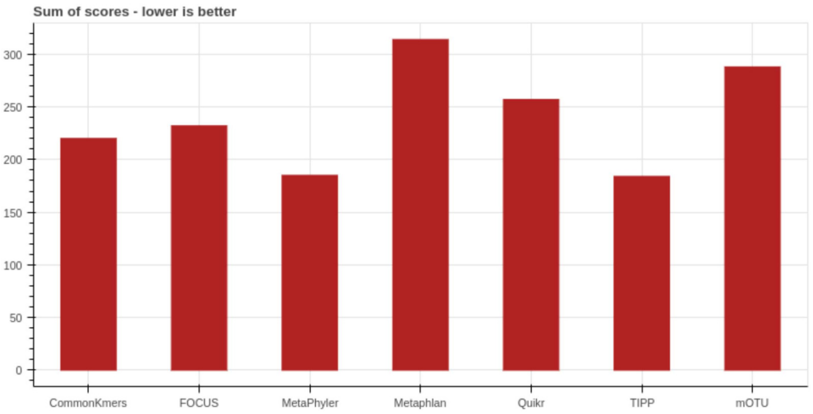

b CAMI II mouse gut dataset

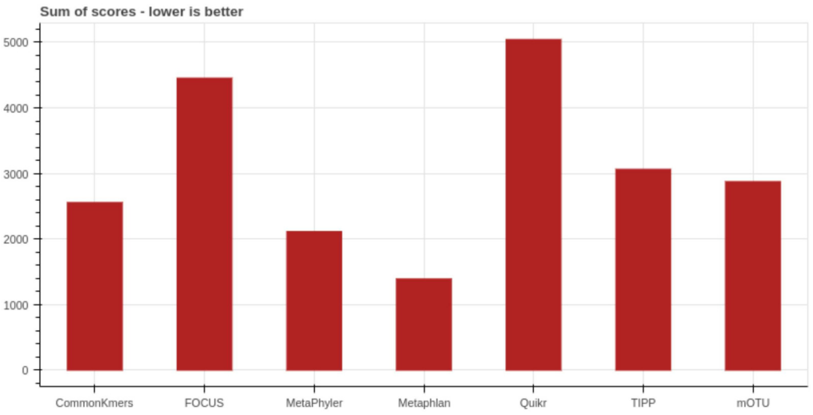

c HMP Mock Community dataset

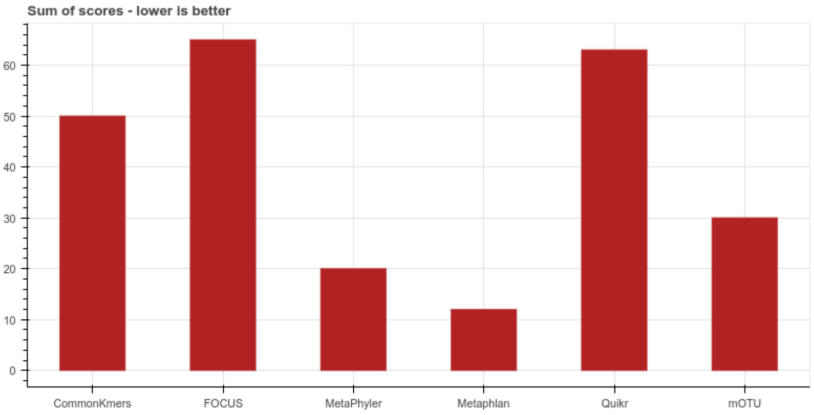

Fig. S11. Sum of scores obtained by profilers as a result of their rankings for the metrics completeness, purity, L1 norm, and weighted UniFrac over all major taxonomic ranks. Scores obtained for each metric are given in Fig. 2d in the main document and Fig. S5e,f above. **a**, **b**, and **c** show the sum of scores on the CAMI I high complexity, the CAMI II mouse gut, and the HMP Mock Community datasets, respectively. For details of the scores computation, see main text.

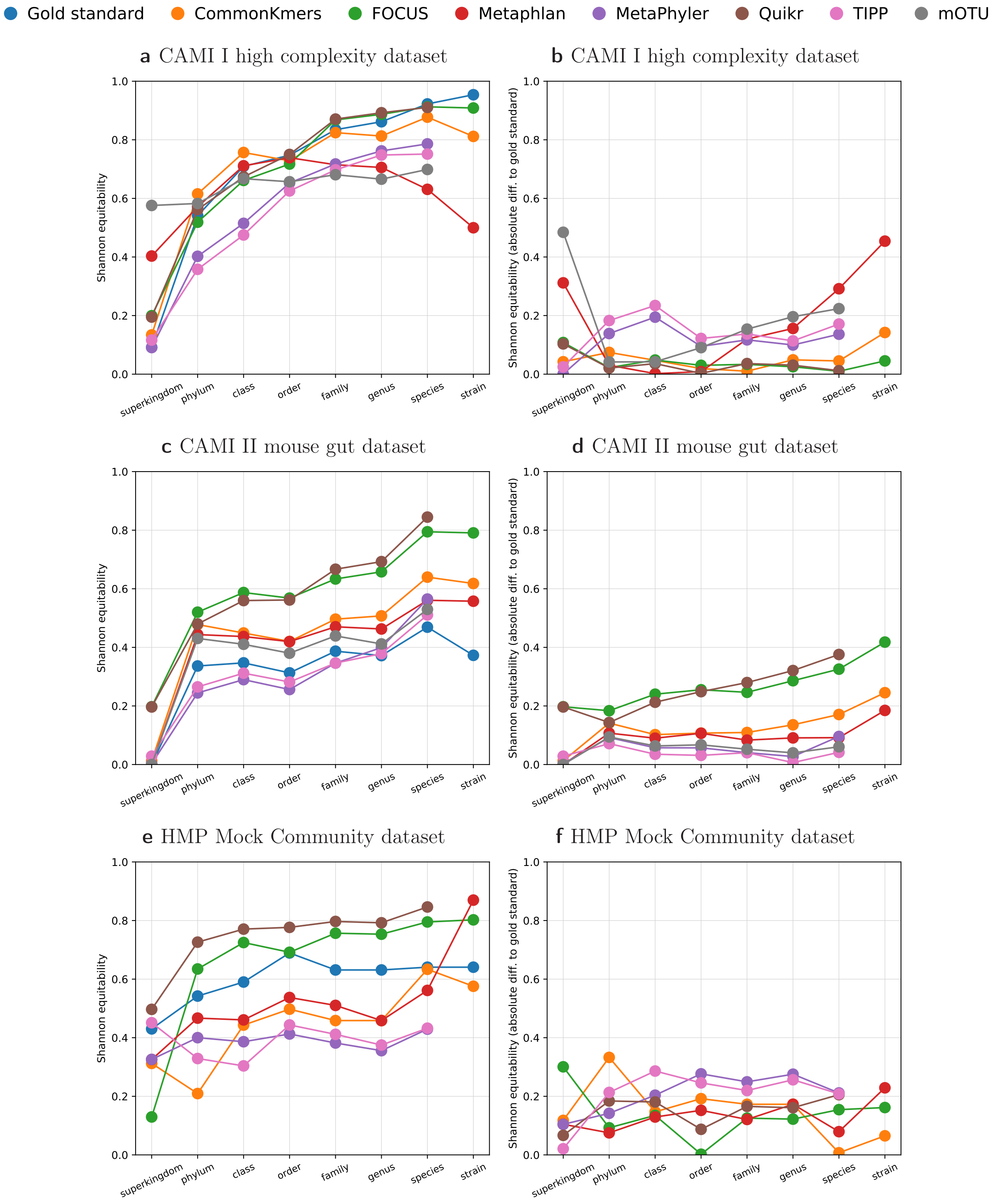

Fig. S12. Shannon equitability computed from the profiling results on the CAMI I high complexity (a), the CAMI II mouse gut (c), and the HMP Mock Community (e) datasets. **b**, **d**, and **f** show the absolute difference in Shannon equitability of each method to the gold standard. The closer the Shannon equitability of the predicted profile by a method to the gold standard, the better it reflects the actual alpha diversity in the gold standard in terms of evenness of the taxa abundances.

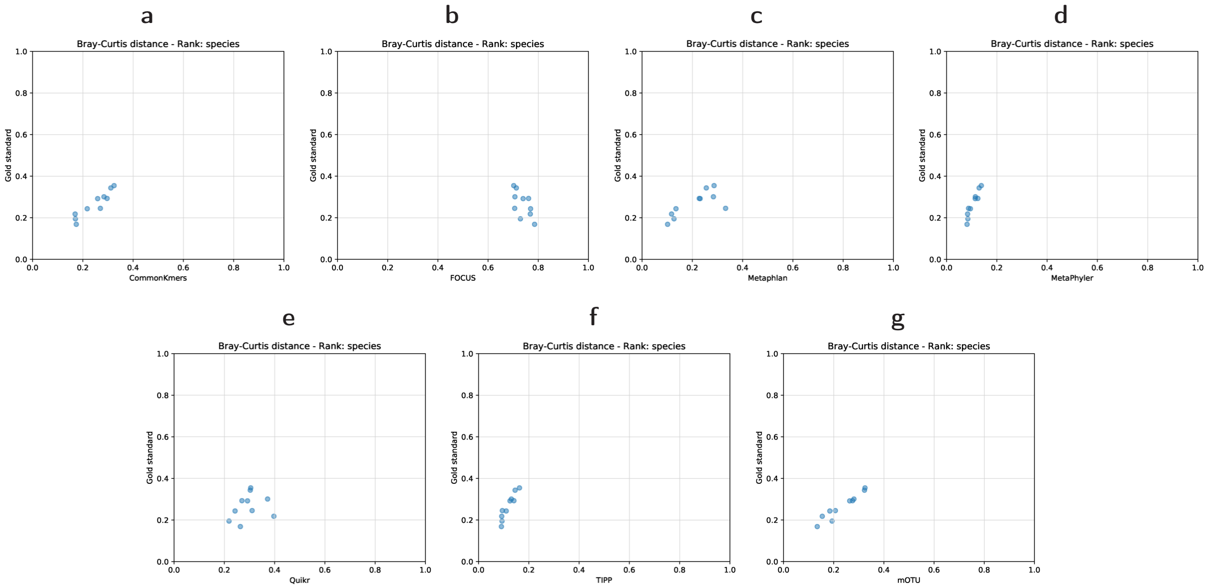

Fig. S13. Scatter plots of Bray-Curtis distances from the profiling results on the CAMI I high complexity dataset. The plots visualize beta diversity at the species level. For each profiling method and plot, a point corresponds to the Bray-Curtis distance between the abundance predictions for a pair of input samples by the method (x-axis) and the Bray-Curtis distance computed for the gold standard for the same pair of samples (y-axis). The closer a point is to the line $x = y$, the more similar the predicted taxa distributions are to the gold standard.
