# Supplementary material for: Assessing taxonomic metagenome profilers with OPAL
Source: Genome Biol. 2019 Mar 4;20:51. doi: 10.1186/s13059-019-1646-y (PMC6398228; doi:10.1186/s13059-019-1646-y)
Supplement: Supplementary file 2 — Instructions for reproducing the comparisons of taxonomic profilers. (PDF 79 kb) [file 13059_2019_1646_MOESM2_ESM.pdf]

**Additional file 2**

# Assessing taxonomic metagenome profilers with OPAL

Fernando Meyer, Andreas Bremges, Peter Belmann, Stefan Janssen, Alice C. McHardy, David Koslicki

### Reproducing OPAL's comparisons of taxonomic profilers on the CAMI I high complexity dataset

• Download the 5 samples of the CAMI I HC dataset from `https://data.cami-challenge.org/participate` and save all files in the same directory. Your directory should contain:

```
RH_S001__insert_270.fq.gz
RH_S002__insert_270.fq.gz
RH_S003__insert_270.fq.gz
RH_S004__insert_270.fq.gz
RH_S005__insert_270.fq.gz
```

• Pull the Bioboxes of profilers:

```
docker pull stefanjanssen/docker_profiling_tools:commonkmers
docker pull stefanjanssen/docker_profiling_tools:focus
docker pull stefanjanssen/docker_profiling_tools:metaphlan2
docker pull stefanjanssen/docker_profiling_tools:metaphyler
docker pull stefanjanssen/docker_profiling_tools:quickr
docker pull stefanjanssen/docker_profiling_tools:tipp
docker pull stefanjanssen/docker_profiling_tools:motu
```

• CommonKmers uses a database that is not stored inside its Biobox. Download it from `https://zenodo.org/record/1749272/files/CommonKmersData.tar.gz?download=1` (DOI: `http://doi.org/10.5281/zenodo.1749272`) and extract the files. Make sure to set the path to the files with option `--volume` for `opal_workflow.py`, as shown below.

```
wget --content-disposition https://zenodo.org/record/1749272/files/CommonKmersData.tar.gz?download=1
tar -xzf CommonKmersData.tar.gz
```

• Install OPAL (`https://github.com/CAMI-challenge/OPAL`).

• OPAL's tool to run Bioboxes of profilers, measure their run time and maximum memory usage, and automatically assess their results is `opal_workflow.py`. To run it, you also need the gold standard file `gs_cami_i_hc.profile` and the Biobox YAML file `biobox_cami_i_hc.yaml`, which are located in the `data` directory of the OPAL GitHub repository (`https://github.com/CAMI-challenge/OPAL/tree/master/data`).

• Run `opal_workflow.py` as follows, modifying the options in red to match your system's paths.

```
./opal_workflow.py \
stefanjanssen/docker_profiling_tools:commonkmers \
stefanjanssen/docker_profiling_tools:focus \
stefanjanssen/docker_profiling_tools:metaphlan2 \
```

```
stefanjanssen/docker_profiling_tools:metaphyler \
stefanjanssen/docker_profiling_tools:quickr \
stefanjanssen/docker_profiling_tools:tipp \
stefanjanssen/docker_profiling_tools:motu \
--labels "CommonKmers, FOCUS, Metaphlan, MetaPhyler, Quikr, TIPP, mOTU" \
--input_dir /path/to/gzipped/fastq/files \
--output_dir /path/to/output_dir \
--yaml /path/to/biobox_cami_i_hc.yaml \
--volume /path/to/CommonKmersData:/exchange/db:ro \
--gold_standard_file /path/to/gs_cami_i_hc.profile \
--plot_abundances \
--desc "1st CAMI Challenge Dataset 3 CAMI high"
```

• The output directory, `output_dir` in this example, will be created if does not exist. It will contain the predictions of all profilers and OPAL's assessments.

### Reproducing OPAL's comparisons of taxonomic profilers on the CAMI II mouse gut dataset

• Download the 64 short-read samples of the CAMI II MG dataset from `https://data.cami-challenge.org/participate`. The files have the same name, but should be located in different sub-directories of the same root directory:

```
2017.12.29_11.37.26_sample_0/reads/anonymous_reads.fq.gz
2017.12.29_11.37.26_sample_1/reads/anonymous_reads.fq.gz
2017.12.29_11.37.26_sample_2/reads/anonymous_reads.fq.gz
...
2017.12.29_11.37.26_sample_63/reads/anonymous_reads.fq.gz
```

• To run `opal_workflow.py`, you also need the gold standard file `gs_cami_i_hc.profile` and the Biobox YAML file `biobox_cami_ii_mg.yaml`, which are located in the `data` directory of the OPAL GitHub repository (`https://github.com/CAMI-challenge/OPAL/tree/master/data`).

• Follow and adapt the other steps given above.

### Reproducing OPAL's comparisons of taxonomic profilers on the HMP MC dataset

• Download the FASTQ file of the HMP MC staggered sample (accession SRX055381) from NCBI SRA (`https://www.ncbi.nlm.nih.gov/sra`) and compress it using gzip. You should have file:

```
SRR172903.fastq.gz
```

• To run `opal_workflow.py`, you also need the gold standard file `gs_hmp_mc.profile` and the Biobox YAML file `biobox_hmp_mc.yaml`, which are located in the `data` directory of the OPAL GitHub repository (`https://github.com/CAMI-challenge/OPAL/tree/master/data`).

• Follow and adapt the other steps given above.
